# Potassium deca­borate monohydrate

**DOI:** 10.1107/S1600536811039109

**Published:** 2011-09-30

**Authors:** Yi-Hong Gao

**Affiliations:** a College of Chemistry & Chemical Engineering, Xianyang Nomal University, Xianyang 712000, People’s Republic of China

## Abstract

In the crystal structure of the title compound, K_2_[B_10_O_14_(OH)_4_]·H_2_O, the polyborate [B_10_O_14_(OH)_4_]^2−^ anions are linked together through their common O atoms, forming a helical chain-like structure. Adjacent chains are further connected into a three-dimensional structure by O—H⋯O hydrogen bonds. The water mol­ecules and potassium cations are located between these chains. Further O—H⋯O hydrogen bonds occur between the anions and the water mol­ecules

## Related literature

For phases previously obtained in the K_2_O—B_2_O_3_—H_2_O system, see: Marezio (1969 ▶); Marezio *et al.* (1963 ▶); Dewey *et al.* (1975 ▶); Salentine (1987 ▶); Touboul *et al.* (2003 ▶); Zhang *et al.* (2005 ▶); Wang *et al.* (2006 ▶); Li *et al.* (2007 ▶). For a closely related structure, (NH_4_)_2_[B_10_O_14_(OH)_4_]·H_2_O, see: Li *et al.* (2003 ▶). For the non-linear optical properties of alkali metal borates, see: Mori *et al.* (1995 ▶).

## Experimental

### 

#### Crystal data

K_2_[B_10_O_14_(OH)_4_]·H_2_O
*M* *_r_* = 496.35
Triclinic, 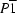

*a* = 7.5612 (7) Å
*b* = 9.2236 (10) Å
*c* = 11.7298 (13) Å
α = 99.038 (6)°
β = 106.595 (6)°
γ = 91.314 (6)°
*V* = 772.26 (14) Å^3^
*Z* = 2
Mo *K*α radiation
μ = 0.72 mm^−1^
*T* = 100 K
0.16 × 0.08 × 0.05 mm

#### Data collection

Bruker APEXII diffractometer
Absorption correction: numerical (*SADABS*, Sheldrick, 2008*a* ▶) *T*_min_ = 0.895, *T*_max_ = 0.962
11219 measured reflections
3148 independent reflections
2141 reflections with *I* > 2σ(*I*)
*R*_int_ = 0.055

#### Refinement

*R*[*F* ^2^ > 2σ(*F* ^2^)] = 0.045
*wR*(*F* ^2^) = 0.113
*S* = 1.00
3148 reflections
298 parameters
5 restraints
All H-atom parameters refined
Δρ_max_ = 0.42 e Å^−3^
Δρ_min_ = −0.46 e Å^−3^

### 

Data collection: *APEX2* (Bruker, 2009 ▶); cell refinement: *SAINT* (Bruker, 2009 ▶); data reduction: *SAINT*; program(s) used to solve structure: *SHELXS97* (Sheldrick, 2008*b* ▶); program(s) used to refine structure: *SHELXL97* (Sheldrick, 2008*b* ▶); molecular graphics: *SHELXTL* (Sheldrick, 2008*b* ▶); software used to prepare material for publication: *SHELXTL*.

## Supplementary Material

Crystal structure: contains datablock(s) I, global. DOI: 10.1107/S1600536811039109/ru2014sup1.cif

Structure factors: contains datablock(s) I. DOI: 10.1107/S1600536811039109/ru2014Isup2.hkl

Additional supplementary materials:  crystallographic information; 3D view; checkCIF report

## Figures and Tables

**Table 1:** Hydrogen-bond geometry (Å, °)

*D*—H⋯*A*	*D*—H	H⋯*A*	*D*⋯*A*	*D*—H⋯*A*
O1—H1⋯O11^i^	0.88 (2)	1.76 (2)	2.599 (3)	159 (3)
O9—H9⋯O18^ii^	0.87 (2)	1.98 (2)	2.797 (3)	157 (3)
O11—H11⋯O19^iii^	0.92 (2)	1.65 (2)	2.553 (3)	167 (3)
O18—H18⋯O5^iv^	0.89 (2)	2.10 (2)	2.940 (3)	157 (3)
O18—H18⋯O12^iv^	0.89 (2)	2.66 (3)	3.193 (3)	119 (3)
O19—H19*A*⋯O6^v^	0.90 (2)	1.79 (3)	2.678 (3)	169 (3)
O19—H19*B*⋯O16^vi^	0.91 (2)	1.79 (3)	2.696 (3)	173 (3)

Symmetry codes: (i) 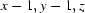; (ii) 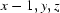; (iii) 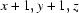; (iv) 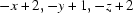; (v) 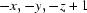; (vi) 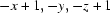.

**Fig. 1.**
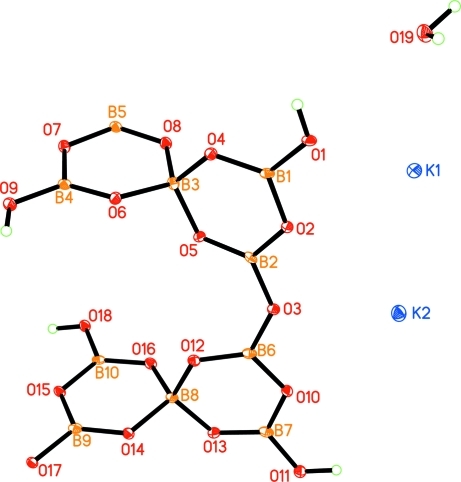
The asymmetric unit structure of title compound. Displacement ellipsoids are drawn at the 30% probability level.

**Fig. 2.**
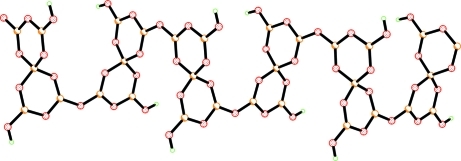
The one-dimensional chain structure constructed by [B10O14(OH)4]2- units. B, O and H atoms are shown as yellow, red and green, respectively.

**Fig. 3.**
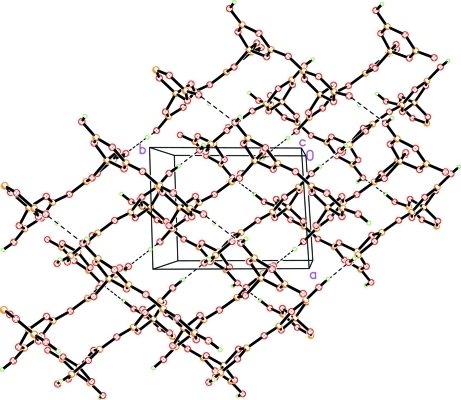
Packing View along the c axis of title compound, showing three-dimensional structure constructed by O—H···O hydrogen bonds, where all potassium cations are omitted for clarity. B, O and H atoms are shown as yellow, red and green, respectively.

## supplementary crystallographic information

### Comment

Boron can form many compounds because of the complexity of the structures involved. In the past several decades, much interest has focused on studies of alkali metals borates because some of these compounds show interesting physical properties, such as nonlinear optical behavior for CsLiB_6_O_10_ (Mori *et al.*, 1995). So far, several phases had been obtained in the K_2_O—B_2_O_3_—H_2_O system (Marezio *et al.*, 1963; Marezio, 1969; Dewey *et al.*, 1975; Salentine,1987; Touboul *et al.*, 2003; Zhang *et al.*, 2005; Wang *et al.*, 2006; Li *et al.*, 2007). In this paper, we describe the synthesis and the crystal structure of a new potassium borate of K_2_[B_10_O_14_(OH)_4_]^.^H_2_O.

Single crystal diffraction has revealed that the title compound crystallizes in the triclinic space group P-1. It is composed of two K_+_ cation and polyborate anion [B_10_O_14_(OH)_4_]_2-_ (Fig.1), which is closely related to the reported compound of (NH_4_)_2_[B_10_O_14_(OH)_4_]^.^H_2_O (Li *et al.*, 2003).

The [B_10_O_14_(OH)_4_]_2-_ anion could be considered as two [B_5_O_7_(OH)_2_]_-_ cluster linked by the common oxygen atom (O3). Each of the [B_5_O_7_(OH)_2_]_-_ cluster consists of two six-membered rings linked by a common BO_4_ tetrahedron. Each six-membered ring consists of one BO_3_ triangle, one BO_2_(OH) triangle and a common BO_4_ tetrahedron. The [B_10_O_14_(OH)_4_]_2-_ units are linked together through common oxygen atoms (O17) to neighboring units, forming a 1-D helical chainlike structure (Fig. 2). Adjacent chains are further connected into a three-dimensional structure by O—H···O hydrogen bonds interactions (Fig.3). Water molecules and potassium ions are located among these chains. In addition, there exist O—H···O hydrogen bonds between the oxygen atoms in polyborate anions and Water molecules (Table 1).

### Experimental

All reagents used in the synthesis were of analytic grade and were used without further purification. A mixture of K_2_TeO_4_ (0.216 g) and H_3_BO_3_ (0.992 g) was sealed in a teflon-lined bomb and heated at 473 K for 5 days and then cooled to room temperature. The resulting colorless and transparent crystals were recovered by washed with deionized water and dried at room temperature.

### Refinement

Hydroxyl and water H atoms were identified from a difference Fourier map and were included in with refined positional parameters.

### Crystal data

**Table:**

H_6_B_10_K_2_O_19_	*Z* = 2
*M**_r_* = 496.35	*F*(000) = 492
Triclinic, *P*1	*D*_x_ = 2.135 Mg m^−^^3^
Hall symbol: -P 1	Mo *K*α radiation, λ = 0.71073 Å
*a* = 7.5612 (7) Å	Cell parameters from 1883 reflections
*b* = 9.2236 (10) Å	θ = 2.6–23.1°
*c* = 11.7298 (13) Å	µ = 0.72 mm^−^^1^
α = 99.038 (6)°	*T* = 100 K
β = 106.595 (6)°	Rod, colorless
γ = 91.314 (6)°	0.16 × 0.08 × 0.05 mm
*V* = 772.26 (14) Å^3^	

### Data collection

**Table:**

Bruker APEXII diffractometer	3148 independent reflections
Radiation source: fine-focus sealed tube	2141 reflections with *I* > 2σ(*I*)
graphite	*R*_int_ = 0.055
Detector resolution: 83.33 pixels mm^-1^	θ_max_ = 26.5°, θ_min_ = 1.8°
combination of ω and φ–scans	*h* = −9→7
Absorption correction: numerical (*SADABS*, Sheldrick, 2008*a*)	*k* = −11→11
*T*_min_ = 0.895, *T*_max_ = 0.962	*l* = −13→14
11219 measured reflections	

### Refinement

**Table:**

Refinement on *F*^2^	Primary atom site location: structure-invariant direct methods
Least-squares matrix: full	Secondary atom site location: difference Fourier map
*R*[*F*^2^ > 2σ(*F*^2^)] = 0.045	Hydrogen site location: inferred from neighbouring sites
*wR*(*F*^2^) = 0.113	All H-atom parameters refined
*S* = 1.00	*w* = 1/[σ^2^(*F*_o_^2^) + (0.0546*P*)^2^] where *P* = (*F*_o_^2^ + 2*F*_c_^2^)/3
3148 reflections	(Δ/σ)_max_ = 0.002
298 parameters	Δρ_max_ = 0.42 e Å^−^^3^
5 restraints	Δρ_min_ = −0.46 e Å^−^^3^

### Special details

**Table:**

Geometry. All e.s.d.'s (except the e.s.d. in the dihedral angle between two l.s. planes) are estimated using the full covariance matrix. The cell e.s.d.'s are taken into account individually in the estimation of e.s.d.'s in distances, angles and torsion angles; correlations between e.s.d.'s in cell parameters are only used when they are defined by crystal symmetry. An approximate (isotropic) treatment of cell e.s.d.'s is used for estimating e.s.d.'s involving l.s. planes.
Refinement. Refinement of *F*^2^ against ALL reflections. The weighted *R*-factor *wR* and goodness of fit *S* are based on *F*^2^, conventional *R*-factors *R* are based on *F*, with *F* set to zero for negative *F*^2^. The threshold expression of *F*^2^ > σ(*F*^2^) is used only for calculating *R*-factors(gt) *etc*. and is not relevant to the choice of reflections for refinement. *R*-factors based on *F*^2^ are statistically about twice as large as those based on *F*, and *R*- factors based on ALL data will be even larger.Hydroxyl and water H atoms were identified from a difference Fourier map and were included in with refined positional parameters. The thermal parameters of these H atoms were tied to that of the oxygen to which they are bonded. Mild O—H distances restraints were applied. All of the H atoms form good H-bonds to nearby O atoms.

### Fractional atomic coordinates and isotropic or equivalent isotropic displacement parameters (Å^2^)

**Table:**

	*x*	*y*	*z*	*U*_iso_*/*U*_eq_	
K1	0.02003 (11)	−0.04398 (8)	0.24158 (6)	0.0193 (2)	
K2	0.57020 (11)	0.25524 (8)	0.28774 (7)	0.0228 (2)	
O1	0.1964 (3)	−0.0442 (2)	0.46853 (19)	0.0157 (5)	
H1	0.132 (4)	−0.108 (3)	0.492 (3)	0.019*	
O2	0.4077 (3)	0.1569 (2)	0.51318 (18)	0.0135 (5)	
O3	0.6155 (3)	0.3561 (2)	0.54323 (18)	0.0133 (5)	
O4	0.3447 (3)	0.0566 (2)	0.67480 (18)	0.0137 (5)	
O5	0.5940 (3)	0.2474 (2)	0.71450 (18)	0.0129 (5)	
O6	0.4224 (3)	0.2380 (2)	0.85610 (18)	0.0135 (5)	
O7	0.6184 (3)	0.1485 (2)	1.02546 (18)	0.0133 (5)	
O8	0.6280 (3)	0.0473 (2)	0.82680 (18)	0.0137 (5)	
O9	0.4452 (3)	0.3433 (2)	1.06130 (19)	0.0158 (5)	
H9	0.372 (4)	0.410 (3)	1.037 (3)	0.019*	
O10	0.8021 (3)	0.5425 (2)	0.51410 (18)	0.0142 (5)	
O11	0.9851 (3)	0.7373 (2)	0.48289 (19)	0.0162 (5)	
H11	0.948 (4)	0.698 (3)	0.4022 (17)	0.019*	
O12	0.7542 (3)	0.5525 (2)	0.70700 (18)	0.0134 (5)	
O13	0.9867 (3)	0.7205 (2)	0.67953 (18)	0.0148 (5)	
O14	0.8285 (3)	0.7940 (2)	0.82488 (18)	0.0129 (5)	
O15	0.9989 (3)	0.7625 (2)	1.02361 (18)	0.0131 (5)	
O16	1.0478 (3)	0.6058 (2)	0.85501 (19)	0.0144 (5)	
O17	0.7994 (3)	0.9505 (2)	1.00052 (18)	0.0122 (5)	
O18	1.2111 (3)	0.5743 (2)	1.04997 (19)	0.0149 (5)	
H18	1.247 (4)	0.615 (3)	1.1280 (17)	0.018*	
O19	−0.0980 (4)	−0.3366 (3)	0.2539 (2)	0.0208 (6)	
H19A	−0.213 (4)	−0.315 (4)	0.214 (3)	0.025*	
H19B	−0.079 (5)	−0.424 (3)	0.213 (3)	0.025*	
B1	0.3158 (5)	0.0559 (4)	0.5553 (3)	0.0130 (8)	
B2	0.5436 (5)	0.2550 (4)	0.5960 (3)	0.0114 (8)	
B3	0.4978 (5)	0.1472 (4)	0.7673 (3)	0.0133 (8)	
B4	0.4912 (5)	0.2461 (4)	0.9766 (3)	0.0130 (8)	
B5	0.6787 (5)	0.0482 (4)	0.9462 (3)	0.0126 (8)	
B6	0.7263 (5)	0.4844 (4)	0.5928 (3)	0.0144 (8)	
B7	0.9241 (5)	0.6669 (4)	0.5601 (3)	0.0145 (8)	
B8	0.9041 (5)	0.6680 (4)	0.7659 (3)	0.0125 (8)	
B9	0.8708 (5)	0.8327 (4)	0.9451 (3)	0.0139 (8)	
B10	1.0838 (5)	0.6471 (4)	0.9752 (3)	0.0131 (8)	

### Atomic displacement parameters (Å^2^)

**Table:**

	*U*^11^	*U*^22^	*U*^33^	*U*^12^	*U*^13^	*U*^23^
K1	0.0252 (5)	0.0164 (4)	0.0155 (4)	−0.0002 (3)	0.0045 (3)	0.0032 (3)
K2	0.0245 (5)	0.0231 (4)	0.0189 (4)	0.0053 (3)	0.0034 (3)	0.0028 (3)
O1	0.0177 (14)	0.0115 (12)	0.0170 (12)	−0.0047 (10)	0.0038 (10)	0.0028 (9)
O2	0.0161 (13)	0.0119 (12)	0.0119 (11)	−0.0017 (10)	0.0038 (10)	0.0015 (9)
O3	0.0137 (13)	0.0117 (12)	0.0138 (11)	−0.0025 (10)	0.0036 (10)	0.0017 (9)
O4	0.0138 (13)	0.0139 (12)	0.0123 (12)	−0.0025 (10)	0.0025 (10)	0.0020 (9)
O5	0.0150 (13)	0.0118 (12)	0.0123 (11)	−0.0014 (10)	0.0044 (10)	0.0029 (9)
O6	0.0152 (13)	0.0134 (12)	0.0129 (12)	0.0023 (10)	0.0050 (10)	0.0031 (9)
O7	0.0124 (13)	0.0140 (12)	0.0136 (12)	0.0031 (10)	0.0040 (10)	0.0015 (9)
O8	0.0143 (13)	0.0132 (12)	0.0132 (12)	0.0028 (10)	0.0038 (10)	0.0016 (9)
O9	0.0181 (14)	0.0137 (12)	0.0169 (12)	0.0052 (10)	0.0065 (10)	0.0030 (10)
O10	0.0174 (14)	0.0122 (12)	0.0134 (12)	−0.0025 (10)	0.0065 (10)	0.0000 (9)
O11	0.0186 (14)	0.0148 (12)	0.0155 (12)	−0.0012 (10)	0.0061 (11)	0.0016 (10)
O12	0.0131 (13)	0.0116 (12)	0.0150 (12)	−0.0032 (9)	0.0045 (10)	0.0010 (9)
O13	0.0171 (14)	0.0125 (12)	0.0150 (12)	−0.0008 (10)	0.0048 (10)	0.0031 (9)
O14	0.0136 (13)	0.0116 (12)	0.0132 (12)	0.0015 (9)	0.0039 (10)	0.0014 (9)
O15	0.0136 (13)	0.0108 (12)	0.0141 (12)	0.0041 (10)	0.0028 (10)	0.0015 (9)
O16	0.0141 (13)	0.0128 (12)	0.0146 (12)	0.0021 (10)	0.0019 (10)	0.0017 (9)
O17	0.0135 (13)	0.0101 (11)	0.0126 (11)	0.0015 (9)	0.0037 (9)	0.0010 (9)
O18	0.0161 (14)	0.0143 (12)	0.0128 (12)	0.0027 (10)	0.0023 (10)	0.0013 (10)
O19	0.0255 (16)	0.0176 (13)	0.0182 (13)	0.0077 (12)	0.0048 (11)	0.0015 (10)
B1	0.012 (2)	0.0091 (18)	0.020 (2)	0.0032 (16)	0.0077 (17)	0.0047 (15)
B2	0.008 (2)	0.0087 (18)	0.018 (2)	0.0039 (15)	0.0052 (16)	−0.0012 (15)
B3	0.012 (2)	0.014 (2)	0.014 (2)	0.0037 (16)	0.0050 (16)	0.0009 (15)
B4	0.012 (2)	0.0109 (19)	0.017 (2)	−0.0034 (16)	0.0061 (16)	0.0010 (15)
B5	0.011 (2)	0.0112 (19)	0.016 (2)	−0.0019 (16)	0.0053 (16)	0.0002 (15)
B6	0.012 (2)	0.0109 (19)	0.020 (2)	0.0038 (16)	0.0038 (17)	0.0052 (16)
B7	0.010 (2)	0.0108 (19)	0.023 (2)	0.0028 (15)	0.0047 (17)	0.0036 (16)
B8	0.011 (2)	0.0099 (19)	0.016 (2)	−0.0008 (15)	0.0042 (16)	0.0014 (15)
B9	0.012 (2)	0.0108 (19)	0.020 (2)	−0.0033 (15)	0.0054 (16)	0.0030 (16)
B10	0.012 (2)	0.0094 (19)	0.017 (2)	−0.0020 (16)	0.0031 (16)	0.0034 (15)

### Geometric parameters (Å, °)

**Table:**

K1—O1	2.615 (2)	O8—B5	1.341 (4)
K1—O17^i^	2.835 (2)	O8—B3	1.469 (4)
K1—O15^i^	2.841 (2)	O8—K1^iii^	2.990 (2)
K1—O19	2.861 (3)	O8—K2^iii^	3.052 (2)
K1—O14^ii^	2.862 (2)	O9—B4	1.358 (4)
K1—O13^ii^	2.988 (2)	O9—K2^viii^	2.808 (2)
K1—O8^iii^	2.990 (2)	O9—H9	0.865 (18)
K1—O4^iv^	3.185 (2)	O10—B6	1.383 (4)
K1—B9^i^	3.335 (4)	O10—B7	1.390 (4)
K1—B9^ii^	3.402 (4)	O11—B7	1.366 (4)
K1—B8^ii^	3.521 (4)	O11—H11	0.918 (18)
K1—B5^iii^	3.589 (4)	O12—B6	1.342 (4)
K1—H1	2.97 (3)	O12—B8	1.472 (4)
K1—H19A	2.95 (4)	O12—K2^ii^	3.069 (2)
K2—O9^v^	2.808 (2)	O13—B7	1.350 (4)
K2—O3	2.913 (2)	O13—B8	1.468 (4)
K2—O14^ii^	2.918 (2)	O13—K1^ii^	2.988 (2)
K2—O4^iii^	3.033 (2)	O13—K2^vi^	3.260 (2)
K2—O8^iii^	3.052 (2)	O14—B9	1.340 (4)
K2—O12^ii^	3.069 (2)	O14—B8	1.469 (4)
K2—O7^v^	3.205 (2)	O14—K1^ii^	2.862 (2)
K2—O13^vi^	3.260 (2)	O14—K2^ii^	2.918 (2)
K2—B4^v^	3.513 (4)	O15—B9	1.383 (4)
K2—B8^ii^	3.568 (4)	O15—B10	1.385 (4)
K2—K1^vii^	4.5268 (12)	O15—K1^ix^	2.840 (2)
O1—B1	1.358 (4)	O16—B10	1.347 (4)
O1—H1	0.881 (18)	O16—B8	1.473 (4)
O2—B1	1.380 (4)	O17—B9	1.379 (4)
O2—B2	1.389 (4)	O17—B5^x^	1.392 (4)
O3—B6	1.378 (4)	O17—K1^ix^	2.835 (2)
O3—B2	1.378 (4)	O18—B10	1.370 (4)
O4—B1	1.355 (4)	O18—H18	0.892 (18)
O4—B3	1.473 (4)	O19—H19A	0.90 (2)
O4—K2^iii^	3.033 (2)	O19—H19B	0.91 (2)
O4—K1^iv^	3.185 (2)	B4—K2^viii^	3.513 (4)
O5—B2	1.346 (4)	B5—O17^xi^	1.392 (4)
O5—B3	1.474 (4)	B5—K1^iii^	3.589 (4)
O6—B4	1.349 (4)	B8—K1^ii^	3.521 (4)
O6—B3	1.483 (4)	B8—K2^ii^	3.567 (4)
O7—B5	1.386 (4)	B9—K1^ix^	3.335 (4)
O7—B4	1.396 (4)	B9—K1^ii^	3.402 (4)
O7—K2^viii^	3.205 (2)		
			
O1—K1—O17^i^	174.90 (7)	B4^v^—K2—B8^ii^	85.73 (9)
O1—K1—O15^i^	133.58 (7)	O9^v^—K2—K1^vii^	102.67 (5)
O17^i^—K1—O15^i^	48.29 (6)	O3—K2—K1^vii^	110.21 (5)
O1—K1—O19	82.07 (7)	O14^ii^—K2—K1^vii^	128.57 (5)
O17^i^—K1—O19	95.19 (7)	O4^iii^—K2—K1^vii^	44.62 (4)
O15^i^—K1—O19	69.42 (7)	O8^iii^—K2—K1^vii^	74.44 (4)
O1—K1—O14^ii^	106.78 (7)	O12^ii^—K2—K1^vii^	174.44 (5)
O17^i^—K1—O14^ii^	76.92 (6)	O7^v^—K2—K1^vii^	59.57 (4)
O15^i^—K1—O14^ii^	95.24 (6)	O13^vi^—K2—K1^vii^	41.24 (4)
O19—K1—O14^ii^	163.93 (7)	B4^v^—K2—K1^vii^	82.73 (7)
O1—K1—O13^ii^	84.58 (7)	B8^ii^—K2—K1^vii^	151.92 (6)
O17^i^—K1—O13^ii^	95.68 (6)	O9^v^—K2—K1	95.28 (5)
O15^i^—K1—O13^ii^	137.05 (6)	O3—K2—K1	92.11 (5)
O19—K1—O13^ii^	148.03 (7)	O14^ii^—K2—K1	33.68 (4)
O14^ii^—K1—O13^ii^	47.83 (6)	O4^iii^—K2—K1	68.36 (5)
O1—K1—O8^iii^	92.13 (7)	O8^iii^—K2—K1	37.13 (4)
O17^i^—K1—O8^iii^	92.85 (6)	O12^ii^—K2—K1	69.44 (4)
O15^i^—K1—O8^iii^	65.98 (6)	O7^v^—K2—K1	98.32 (4)
O19—K1—O8^iii^	109.80 (7)	O13^vi^—K2—K1	148.91 (4)
O14^ii^—K1—O8^iii^	57.44 (6)	B4^v^—K2—K1	94.67 (6)
O13^ii^—K1—O8^iii^	99.57 (6)	B8^ii^—K2—K1	47.09 (6)
O1—K1—O4^iv^	85.11 (7)	K1^vii^—K2—K1	108.49 (2)
O17^i^—K1—O4^iv^	89.86 (6)	B1—O1—K1	133.0 (2)
O15^i^—K1—O4^iv^	114.24 (6)	B1—O1—H1	118 (2)
O19—K1—O4^iv^	67.25 (6)	K1—O1—H1	105 (2)
O14^ii^—K1—O4^iv^	125.91 (6)	B1—O2—B2	118.7 (3)
O13^ii^—K1—O4^iv^	82.81 (6)	B6—O3—B2	131.3 (3)
O8^iii^—K1—O4^iv^	176.19 (6)	B6—O3—K2	114.9 (2)
O1—K1—B9^i^	157.71 (9)	B2—O3—K2	113.08 (18)
O17^i^—K1—B9^i^	24.12 (8)	B1—O4—B3	122.0 (3)
O15^i^—K1—B9^i^	24.22 (8)	B1—O4—K2^iii^	105.55 (18)
O19—K1—B9^i^	82.81 (8)	B3—O4—K2^iii^	103.33 (17)
O14^ii^—K1—B9^i^	84.64 (8)	B1—O4—K1^iv^	115.2 (2)
O13^ii^—K1—B9^i^	116.44 (8)	B3—O4—K1^iv^	111.89 (18)
O8^iii^—K1—B9^i^	77.75 (8)	K2^iii^—O4—K1^iv^	93.40 (6)
O4^iv^—K1—B9^i^	103.92 (8)	B2—O5—B3	123.1 (3)
O1—K1—B9^ii^	127.37 (8)	B4—O6—B3	123.6 (3)
O17^i^—K1—B9^ii^	56.94 (8)	B5—O7—B4	117.8 (3)
O15^i^—K1—B9^ii^	73.78 (8)	B5—O7—K2^viii^	150.4 (2)
O19—K1—B9^ii^	143.12 (8)	B4—O7—K2^viii^	90.76 (18)
O14^ii^—K1—B9^ii^	22.67 (7)	B5—O8—B3	123.5 (3)
O13^ii^—K1—B9^ii^	65.68 (8)	B5—O8—K1^iii^	105.5 (2)
O8^iii^—K1—B9^ii^	54.80 (8)	B3—O8—K1^iii^	113.16 (19)
O4^iv^—K1—B9^ii^	129.01 (8)	B5—O8—K2^iii^	105.48 (19)
B9^i^—K1—B9^ii^	62.00 (11)	B3—O8—K2^iii^	102.58 (18)
O1—K1—B8^ii^	98.97 (8)	K1^iii^—O8—K2^iii^	104.84 (6)
O17^i^—K1—B8^ii^	83.04 (7)	B4—O9—K2^viii^	110.0 (2)
O15^i^—K1—B8^ii^	115.06 (8)	B4—O9—H9	118 (2)
O19—K1—B8^ii^	170.63 (8)	K2^viii^—O9—H9	131 (2)
O14^ii^—K1—B8^ii^	23.88 (7)	B6—O10—B7	117.7 (3)
O13^ii^—K1—B8^ii^	24.34 (7)	B7—O11—H11	118 (2)
O8^iii^—K1—B8^ii^	79.52 (8)	B6—O12—B8	122.0 (3)
O4^iv^—K1—B8^ii^	103.49 (7)	B6—O12—K2^ii^	107.8 (2)
B9^i^—K1—B8^ii^	98.62 (9)	B8—O12—K2^ii^	97.29 (18)
B9^ii^—K1—B8^ii^	41.76 (8)	B7—O13—B8	121.6 (3)
O1—K1—B5^iii^	113.22 (8)	B7—O13—K1^ii^	117.81 (19)
O17^i^—K1—B5^iii^	71.76 (7)	B8—O13—K1^ii^	98.63 (17)
O15^i^—K1—B5^iii^	51.42 (7)	B7—O13—K2^vi^	99.5 (2)
O19—K1—B5^iii^	111.08 (8)	B8—O13—K2^vi^	124.05 (19)
O14^ii^—K1—B5^iii^	53.30 (7)	K1^ii^—O13—K2^vi^	92.77 (6)
O13^ii^—K1—B5^iii^	100.87 (7)	B9—O14—B8	123.0 (3)
O8^iii^—K1—B5^iii^	21.09 (7)	B9—O14—K1^ii^	101.92 (19)
O4^iv^—K1—B5^iii^	161.47 (7)	B8—O14—K1^ii^	104.07 (18)
B9^i^—K1—B5^iii^	58.07 (9)	B9—O14—K2^ii^	111.8 (2)
B9^ii^—K1—B5^iii^	41.61 (9)	B8—O14—K2^ii^	103.88 (18)
B8^ii^—K1—B5^iii^	77.18 (8)	K1^ii^—O14—K2^ii^	111.90 (7)
O1—K1—H1	16.6 (4)	B9—O15—B10	118.2 (3)
O17^i^—K1—H1	158.9 (5)	B9—O15—K1^ix^	98.35 (19)
O15^i^—K1—H1	128.2 (6)	B10—O15—K1^ix^	142.67 (19)
O19—K1—H1	67.4 (5)	B10—O16—B8	123.4 (3)
O14^ii^—K1—H1	122.9 (5)	B9—O17—B5^x^	127.9 (3)
O13^ii^—K1—H1	94.0 (6)	B9—O17—K1^ix^	98.70 (19)
O8^iii^—K1—H1	104.0 (6)	B5^x^—O17—K1^ix^	132.52 (19)
O4^iv^—K1—H1	72.8 (6)	B10—O18—H18	116 (2)
B9^i^—K1—H1	149.0 (5)	K1—O19—H19A	87 (2)
B9^ii^—K1—H1	143.9 (5)	K1—O19—H19B	129 (2)
B8^ii^—K1—H1	112.2 (5)	H19A—O19—H19B	106 (3)
B5^iii^—K1—H1	124.6 (6)	O4—B1—O1	122.8 (3)
O1—K1—H19A	94.6 (6)	O4—B1—O2	122.0 (3)
O17^i^—K1—H19A	81.8 (6)	O1—B1—O2	115.2 (3)
O15^i^—K1—H19A	68.3 (7)	O5—B2—O3	125.8 (3)
O19—K1—H19A	17.8 (5)	O5—B2—O2	121.4 (3)
O14^ii^—K1—H19A	158.6 (6)	O3—B2—O2	112.9 (3)
O13^ii^—K1—H19A	138.3 (6)	O8—B3—O4	107.8 (3)
O8^iii^—K1—H19A	122.1 (6)	O8—B3—O5	109.8 (3)
O4^iv^—K1—H19A	55.7 (6)	O4—B3—O5	111.9 (3)
B9^i^—K1—H19A	74.9 (6)	O8—B3—O6	110.3 (3)
B9^ii^—K1—H19A	136.6 (6)	O4—B3—O6	109.0 (3)
B8^ii^—K1—H19A	154.1 (5)	O5—B3—O6	108.0 (3)
B5^iii^—K1—H19A	117.3 (7)	O6—B4—O9	125.4 (3)
H1—K1—H19A	78.5 (7)	O6—B4—O7	121.1 (3)
O9^v^—K2—O3	141.92 (7)	O9—B4—O7	113.5 (3)
O9^v^—K2—O14^ii^	65.70 (6)	O6—B4—K2^viii^	167.7 (2)
O3—K2—O14^ii^	105.21 (6)	O9—B4—K2^viii^	48.70 (16)
O9^v^—K2—O4^iii^	124.58 (6)	O7—B4—K2^viii^	65.83 (16)
O3—K2—O4^iii^	92.81 (6)	O8—B5—O7	122.7 (3)
O14^ii^—K2—O4^iii^	98.79 (6)	O8—B5—O17^xi^	122.6 (3)
O9^v^—K2—O8^iii^	88.70 (6)	O7—B5—O17^xi^	114.7 (3)
O3—K2—O8^iii^	117.87 (6)	O8—B5—K1^iii^	53.38 (16)
O14^ii^—K2—O8^iii^	56.20 (6)	O7—B5—K1^iii^	138.7 (2)
O4^iii^—K2—O8^iii^	45.99 (6)	O17^xi^—B5—K1^iii^	83.00 (19)
O9^v^—K2—O12^ii^	72.67 (6)	O12—B6—O3	123.4 (3)
O3—K2—O12^ii^	75.21 (6)	O12—B6—O10	121.7 (3)
O14^ii^—K2—O12^ii^	47.02 (6)	O3—B6—O10	114.8 (3)
O4^iii^—K2—O12^ii^	135.50 (6)	O13—B7—O11	118.4 (3)
O8^iii^—K2—O12^ii^	102.11 (6)	O13—B7—O10	122.2 (3)
O9^v^—K2—O7^v^	44.43 (6)	O11—B7—O10	119.4 (3)
O3—K2—O7^v^	167.31 (6)	O13—B8—O14	107.9 (3)
O14^ii^—K2—O7^v^	87.46 (6)	O13—B8—O12	112.3 (3)
O4^iii^—K2—O7^v^	84.39 (6)	O14—B8—O12	108.8 (3)
O8^iii^—K2—O7^v^	68.47 (6)	O13—B8—O16	109.0 (3)
O12^ii^—K2—O7^v^	115.24 (6)	O14—B8—O16	111.0 (3)
O9^v^—K2—O13^vi^	98.63 (7)	O12—B8—O16	107.9 (3)
O3—K2—O13^vi^	93.83 (6)	O13—B8—K1^ii^	57.03 (15)
O14^ii^—K2—O13^vi^	160.94 (6)	O14—B8—K1^ii^	52.05 (14)
O4^iii^—K2—O13^vi^	80.88 (6)	O12—B8—K1^ii^	136.1 (2)
O8^iii^—K2—O13^vi^	115.44 (6)	O16—B8—K1^ii^	115.82 (19)
O12^ii^—K2—O13^vi^	141.42 (6)	O13—B8—K2^ii^	112.4 (2)
O7^v^—K2—O13^vi^	73.52 (6)	O14—B8—K2^ii^	52.56 (15)
O9^v^—K2—B4^v^	21.30 (7)	O12—B8—K2^ii^	58.56 (15)
O3—K2—B4^v^	162.61 (8)	O16—B8—K2^ii^	138.4 (2)
O14^ii^—K2—B4^v^	72.97 (8)	K1^ii^—B8—K2^ii^	85.00 (8)
O4^iii^—K2—B4^v^	104.57 (7)	O14—B9—O17	123.1 (3)
O8^iii^—K2—B4^v^	76.05 (7)	O14—B9—O15	122.4 (3)
O12^ii^—K2—B4^v^	92.23 (8)	O17—B9—O15	114.4 (3)
O7^v^—K2—B4^v^	23.41 (7)	O14—B9—K1^ix^	172.8 (2)
O13^vi^—K2—B4^v^	88.60 (8)	O17—B9—K1^ix^	57.17 (16)
O9^v^—K2—B8^ii^	71.35 (8)	O15—B9—K1^ix^	57.43 (16)
O3—K2—B8^ii^	87.22 (7)	O14—B9—K1^ii^	55.40 (16)
O14^ii^—K2—B8^ii^	23.57 (7)	O17—B9—K1^ii^	90.79 (19)
O4^iii^—K2—B8^ii^	115.36 (8)	O15—B9—K1^ii^	123.9 (2)
O8^iii^—K2—B8^ii^	77.97 (7)	K1^ix^—B9—K1^ii^	118.00 (11)
O12^ii^—K2—B8^ii^	24.15 (7)	O16—B10—O18	118.4 (3)
O7^v^—K2—B8^ii^	105.18 (7)	O16—B10—O15	121.5 (3)
O13^vi^—K2—B8^ii^	163.68 (7)	O18—B10—O15	120.0 (3)
			
O1—K1—K2—O9^v^	−171.23 (7)	B8^ii^—K2—O3—B2	75.7 (2)
O17^i^—K1—K2—O9^v^	6.68 (7)	K1^vii^—K2—O3—B2	−81.7 (2)
O15^i^—K1—K2—O9^v^	56.06 (7)	K1—K2—O3—B2	28.9 (2)
O19—K1—K2—O9^v^	126.23 (9)	B3—O4—B1—O1	168.5 (3)
O14^ii^—K1—K2—O9^v^	−27.88 (9)	K2^iii^—O4—B1—O1	51.4 (4)
O13^ii^—K1—K2—O9^v^	−81.77 (7)	K1^iv^—O4—B1—O1	−50.1 (4)
O8^iii^—K1—K2—O9^v^	80.80 (8)	B3—O4—B1—O2	−10.9 (5)
O4^iv^—K1—K2—O9^v^	−105.08 (9)	K2^iii^—O4—B1—O2	−128.0 (3)
B9^i^—K1—K2—O9^v^	31.46 (8)	K1^iv^—O4—B1—O2	130.5 (3)
B9^ii^—K1—K2—O9^v^	−1.60 (10)	K1—O1—B1—O4	157.5 (2)
B8^ii^—K1—K2—O9^v^	−58.36 (9)	K1—O1—B1—O2	−23.0 (4)
B5^iii^—K1—K2—O9^v^	54.42 (9)	B2—O2—B1—O4	4.9 (5)
O1—K1—K2—O3	−28.62 (7)	B2—O2—B1—O1	−174.6 (3)
O17^i^—K1—K2—O3	149.28 (6)	B3—O5—B2—O3	173.0 (3)
O15^i^—K1—K2—O3	−161.33 (6)	B3—O5—B2—O2	−6.7 (5)
O19—K1—K2—O3	−91.17 (9)	B6—O3—B2—O5	−12.3 (6)
O14^ii^—K1—K2—O3	114.72 (9)	K2—O3—B2—O5	156.9 (3)
O13^ii^—K1—K2—O3	60.83 (6)	B6—O3—B2—O2	167.4 (3)
O8^iii^—K1—K2—O3	−136.60 (8)	K2—O3—B2—O2	−23.3 (3)
O4^iv^—K1—K2—O3	37.52 (9)	B1—O2—B2—O5	3.9 (4)
B9^i^—K1—K2—O3	174.06 (8)	B1—O2—B2—O3	−175.8 (3)
B9^ii^—K1—K2—O3	141.00 (10)	B5—O8—B3—O4	−123.3 (3)
B8^ii^—K1—K2—O3	84.24 (9)	K1^iii^—O8—B3—O4	107.5 (2)
B5^iii^—K1—K2—O3	−162.98 (9)	K2^iii^—O8—B3—O4	−4.9 (3)
O1—K1—K2—O14^ii^	−143.35 (9)	B5—O8—B3—O5	114.5 (3)
O17^i^—K1—K2—O14^ii^	34.55 (9)	K1^iii^—O8—B3—O5	−14.7 (3)
O15^i^—K1—K2—O14^ii^	83.94 (9)	K2^iii^—O8—B3—O5	−127.1 (2)
O19—K1—K2—O14^ii^	154.10 (11)	B5—O8—B3—O6	−4.4 (4)
O13^ii^—K1—K2—O14^ii^	−53.89 (9)	K1^iii^—O8—B3—O6	−133.6 (2)
O8^iii^—K1—K2—O14^ii^	108.68 (10)	K2^iii^—O8—B3—O6	114.0 (2)
O4^iv^—K1—K2—O14^ii^	−77.20 (10)	B1—O4—B3—O8	−113.3 (3)
B9^i^—K1—K2—O14^ii^	59.34 (10)	K2^iii^—O4—B3—O8	5.0 (3)
B9^ii^—K1—K2—O14^ii^	26.28 (11)	K1^iv^—O4—B3—O8	104.2 (2)
B8^ii^—K1—K2—O14^ii^	−30.49 (10)	B1—O4—B3—O5	7.6 (4)
B5^iii^—K1—K2—O14^ii^	82.30 (11)	K2^iii^—O4—B3—O5	125.8 (2)
O1—K1—K2—O4^iii^	63.57 (7)	K1^iv^—O4—B3—O5	−134.9 (2)
O17^i^—K1—K2—O4^iii^	−118.53 (6)	B1—O4—B3—O6	126.9 (3)
O15^i^—K1—K2—O4^iii^	−69.14 (6)	K2^iii^—O4—B3—O6	−114.8 (2)
O19—K1—K2—O4^iii^	1.02 (9)	K1^iv^—O4—B3—O6	−15.6 (3)
O14^ii^—K1—K2—O4^iii^	−153.08 (9)	B2—O5—B3—O8	120.7 (3)
O13^ii^—K1—K2—O4^iii^	153.02 (6)	B2—O5—B3—O4	1.0 (4)
O8^iii^—K1—K2—O4^iii^	−44.41 (8)	B2—O5—B3—O6	−118.9 (3)
O4^iv^—K1—K2—O4^iii^	129.71 (10)	B4—O6—B3—O8	11.5 (4)
B9^i^—K1—K2—O4^iii^	−93.75 (8)	B4—O6—B3—O4	129.8 (3)
B9^ii^—K1—K2—O4^iii^	−126.81 (10)	B4—O6—B3—O5	−108.5 (3)
B8^ii^—K1—K2—O4^iii^	176.43 (9)	B3—O6—B4—O9	169.7 (3)
B5^iii^—K1—K2—O4^iii^	−70.79 (9)	B3—O6—B4—O7	−11.6 (5)
O1—K1—K2—O8^iii^	107.97 (9)	B3—O6—B4—K2^viii^	−132.9 (10)
O17^i^—K1—K2—O8^iii^	−74.12 (8)	K2^viii^—O9—B4—O6	166.1 (3)
O15^i^—K1—K2—O8^iii^	−24.74 (8)	K2^viii^—O9—B4—O7	−12.7 (3)
O19—K1—K2—O8^iii^	45.42 (10)	B5—O7—B4—O6	3.3 (5)
O14^ii^—K1—K2—O8^iii^	−108.68 (10)	K2^viii^—O7—B4—O6	−168.5 (3)
O13^ii^—K1—K2—O8^iii^	−162.57 (8)	B5—O7—B4—O9	−177.9 (3)
O4^iv^—K1—K2—O8^iii^	174.12 (10)	K2^viii^—O7—B4—O9	10.4 (3)
B9^i^—K1—K2—O8^iii^	−49.34 (9)	B5—O7—B4—K2^viii^	171.7 (3)
B9^ii^—K1—K2—O8^iii^	−82.40 (11)	B3—O8—B5—O7	−2.9 (5)
B8^ii^—K1—K2—O8^iii^	−139.17 (10)	K1^iii^—O8—B5—O7	129.4 (3)
B5^iii^—K1—K2—O8^iii^	−26.38 (10)	K2^iii^—O8—B5—O7	−119.9 (3)
O1—K1—K2—O12^ii^	−101.97 (7)	B3—O8—B5—O17^xi^	178.6 (3)
O17^i^—K1—K2—O12^ii^	75.94 (6)	K1^iii^—O8—B5—O17^xi^	−49.1 (4)
O15^i^—K1—K2—O12^ii^	125.32 (6)	K2^iii^—O8—B5—O17^xi^	61.6 (3)
O19—K1—K2—O12^ii^	−164.51 (9)	B3—O8—B5—K1^iii^	−132.3 (3)
O14^ii^—K1—K2—O12^ii^	41.38 (8)	K2^iii^—O8—B5—K1^iii^	110.65 (13)
O13^ii^—K1—K2—O12^ii^	−12.51 (6)	B4—O7—B5—O8	3.9 (5)
O8^iii^—K1—K2—O12^ii^	150.06 (8)	K2^viii^—O7—B5—O8	166.9 (2)
O4^iv^—K1—K2—O12^ii^	−35.82 (8)	B4—O7—B5—O17^xi^	−177.5 (3)
B9^i^—K1—K2—O12^ii^	100.72 (8)	K2^viii^—O7—B5—O17^xi^	−14.5 (6)
B9^ii^—K1—K2—O12^ii^	67.66 (9)	B4—O7—B5—K1^iii^	73.8 (4)
B8^ii^—K1—K2—O12^ii^	10.89 (9)	K2^viii^—O7—B5—K1^iii^	−123.2 (3)
B5^iii^—K1—K2—O12^ii^	123.68 (9)	B8—O12—B6—O3	165.6 (3)
O1—K1—K2—O7^v^	144.12 (7)	K2^ii^—O12—B6—O3	−83.5 (3)
O17^i^—K1—K2—O7^v^	−37.98 (6)	B8—O12—B6—O10	−17.2 (5)
O15^i^—K1—K2—O7^v^	11.41 (6)	K2^ii^—O12—B6—O10	93.8 (3)
O19—K1—K2—O7^v^	81.57 (9)	B2—O3—B6—O12	−15.1 (6)
O14^ii^—K1—K2—O7^v^	−72.53 (8)	K2—O3—B6—O12	175.8 (2)
O13^ii^—K1—K2—O7^v^	−126.43 (6)	B2—O3—B6—O10	167.5 (3)
O8^iii^—K1—K2—O7^v^	36.14 (8)	K2—O3—B6—O10	−1.6 (4)
O4^iv^—K1—K2—O7^v^	−149.74 (8)	B7—O10—B6—O12	6.3 (5)
B9^i^—K1—K2—O7^v^	−13.20 (8)	B7—O10—B6—O3	−176.2 (3)
B9^ii^—K1—K2—O7^v^	−46.26 (9)	B8—O13—B7—O11	169.1 (3)
B8^ii^—K1—K2—O7^v^	−103.02 (9)	K1^ii^—O13—B7—O11	47.5 (4)
B5^iii^—K1—K2—O7^v^	9.76 (9)	K2^vi^—O13—B7—O11	−50.7 (3)
O1—K1—K2—O13^vi^	72.35 (10)	B8—O13—B7—O10	−11.6 (5)
O17^i^—K1—K2—O13^vi^	−109.75 (9)	K1^ii^—O13—B7—O10	−133.2 (3)
O15^i^—K1—K2—O13^vi^	−60.36 (9)	K2^vi^—O13—B7—O10	128.6 (3)
O19—K1—K2—O13^vi^	9.80 (12)	B6—O10—B7—O13	8.2 (5)
O14^ii^—K1—K2—O13^vi^	−144.30 (11)	B6—O10—B7—O11	−172.5 (3)
O13^ii^—K1—K2—O13^vi^	161.81 (12)	B7—O13—B8—O14	−118.7 (3)
O8^iii^—K1—K2—O13^vi^	−35.62 (10)	K1^ii^—O13—B8—O14	11.6 (3)
O4^iv^—K1—K2—O13^vi^	138.49 (10)	K2^vi^—O13—B8—O14	110.9 (2)
B9^i^—K1—K2—O13^vi^	−84.97 (11)	B7—O13—B8—O12	1.2 (4)
B9^ii^—K1—K2—O13^vi^	−118.03 (12)	K1^ii^—O13—B8—O12	131.5 (2)
B8^ii^—K1—K2—O13^vi^	−174.79 (12)	K2^vi^—O13—B8—O12	−129.2 (2)
B5^iii^—K1—K2—O13^vi^	−62.00 (11)	B7—O13—B8—O16	120.7 (3)
O1—K1—K2—B4^v^	167.40 (8)	K1^ii^—O13—B8—O16	−109.0 (2)
O17^i^—K1—K2—B4^v^	−14.70 (8)	K2^vi^—O13—B8—O16	−9.7 (3)
O15^i^—K1—K2—B4^v^	34.69 (8)	B7—O13—B8—K1^ii^	−130.3 (3)
O19—K1—K2—B4^v^	104.85 (10)	K2^vi^—O13—B8—K1^ii^	99.29 (16)
O14^ii^—K1—K2—B4^v^	−49.25 (10)	B7—O13—B8—K2^ii^	−62.6 (3)
O13^ii^—K1—K2—B4^v^	−103.14 (8)	K1^ii^—O13—B8—K2^ii^	67.70 (15)
O8^iii^—K1—K2—B4^v^	59.43 (9)	K2^vi^—O13—B8—K2^ii^	166.99 (8)
O4^iv^—K1—K2—B4^v^	−126.46 (9)	B9—O14—B8—O13	−127.0 (3)
B9^i^—K1—K2—B4^v^	10.08 (9)	K1^ii^—O14—B8—O13	−12.4 (3)
B9^ii^—K1—K2—B4^v^	−22.97 (11)	K2^ii^—O14—B8—O13	104.9 (2)
B8^ii^—K1—K2—B4^v^	−79.74 (10)	B9—O14—B8—O12	110.9 (3)
B5^iii^—K1—K2—B4^v^	33.05 (10)	K1^ii^—O14—B8—O12	−134.4 (2)
O1—K1—K2—B8^ii^	−112.86 (10)	K2^ii^—O14—B8—O12	−17.1 (3)
O17^i^—K1—K2—B8^ii^	65.04 (9)	B9—O14—B8—O16	−7.6 (4)
O15^i^—K1—K2—B8^ii^	114.43 (9)	K1^ii^—O14—B8—O16	107.0 (2)
O19—K1—K2—B8^ii^	−175.41 (11)	K2^ii^—O14—B8—O16	−135.7 (2)
O14^ii^—K1—K2—B8^ii^	30.49 (10)	B9—O14—B8—K1^ii^	−114.6 (3)
O13^ii^—K1—K2—B8^ii^	−23.40 (9)	K2^ii^—O14—B8—K1^ii^	117.26 (12)
O8^iii^—K1—K2—B8^ii^	139.17 (10)	B9—O14—B8—K2^ii^	128.1 (3)
O4^iv^—K1—K2—B8^ii^	−46.71 (11)	K1^ii^—O14—B8—K2^ii^	−117.26 (12)
B9^i^—K1—K2—B8^ii^	89.82 (11)	B6—O12—B8—O13	12.9 (4)
B9^ii^—K1—K2—B8^ii^	56.77 (11)	K2^ii^—O12—B8—O13	−103.4 (2)
B5^iii^—K1—K2—B8^ii^	112.79 (11)	B6—O12—B8—O14	132.3 (3)
O1—K1—K2—K1^vii^	83.51 (6)	K2^ii^—O12—B8—O14	15.9 (2)
O17^i^—K1—K2—K1^vii^	−98.58 (5)	B6—O12—B8—O16	−107.2 (3)
O15^i^—K1—K2—K1^vii^	−49.20 (5)	K2^ii^—O12—B8—O16	136.4 (2)
O19—K1—K2—K1^vii^	20.96 (9)	B6—O12—B8—K1^ii^	77.9 (4)
O14^ii^—K1—K2—K1^vii^	−133.14 (8)	K2^ii^—O12—B8—K1^ii^	−38.4 (3)
O13^ii^—K1—K2—K1^vii^	172.97 (5)	B6—O12—B8—K2^ii^	116.4 (3)
O8^iii^—K1—K2—K1^vii^	−24.46 (7)	B10—O16—B8—O13	127.4 (3)
O4^iv^—K1—K2—K1^vii^	149.66 (7)	B10—O16—B8—O14	8.7 (4)
B9^i^—K1—K2—K1^vii^	−73.80 (7)	B10—O16—B8—O12	−110.5 (3)
B9^ii^—K1—K2—K1^vii^	−106.86 (9)	B10—O16—B8—K1^ii^	65.6 (3)
B8^ii^—K1—K2—K1^vii^	−163.63 (8)	B10—O16—B8—K2^ii^	−48.0 (4)
B5^iii^—K1—K2—K1^vii^	−50.84 (8)	B8—O14—B9—O17	−179.7 (3)
O17^i^—K1—O1—B1	−125.6 (7)	K1^ii^—O14—B9—O17	64.6 (3)
O15^i^—K1—O1—B1	125.2 (3)	K2^ii^—O14—B9—O17	−55.1 (4)
O19—K1—O1—B1	176.7 (3)	B8—O14—B9—O15	4.2 (5)
O14^ii^—K1—O1—B1	10.5 (3)	K1^ii^—O14—B9—O15	−111.5 (3)
O13^ii^—K1—O1—B1	−32.4 (3)	K2^ii^—O14—B9—O15	128.9 (3)
O8^iii^—K1—O1—B1	67.0 (3)	B8—O14—B9—K1^ix^	90.4 (19)
O4^iv^—K1—O1—B1	−115.6 (3)	K1^ii^—O14—B9—K1^ix^	−25 (2)
B9^i^—K1—O1—B1	129.0 (3)	K2^ii^—O14—B9—K1^ix^	−145.0 (19)
B9^ii^—K1—O1—B1	21.2 (3)	B8—O14—B9—K1^ii^	115.7 (3)
B8^ii^—K1—O1—B1	−12.7 (3)	K2^ii^—O14—B9—K1^ii^	−119.68 (15)
B5^iii^—K1—O1—B1	67.1 (3)	B5^x^—O17—B9—O14	−1.0 (5)
O9^v^—K2—O3—B6	−58.7 (2)	K1^ix^—O17—B9—O14	−171.4 (3)
O14^ii^—K2—O3—B6	−128.5 (2)	B5^x^—O17—B9—O15	175.3 (3)
O4^iii^—K2—O3—B6	131.6 (2)	K1^ix^—O17—B9—O15	4.9 (3)
O8^iii^—K2—O3—B6	172.0 (2)	B5^x^—O17—B9—K1^ix^	170.3 (3)
O12^ii^—K2—O3—B6	−91.9 (2)	B5^x^—O17—B9—K1^ii^	47.0 (3)
O7^v^—K2—O3—B6	54.7 (4)	K1^ix^—O17—B9—K1^ii^	−123.35 (8)
O13^vi^—K2—O3—B6	50.5 (2)	B10—O15—B9—O14	−0.8 (5)
B4^v^—K2—O3—B6	−47.0 (4)	K1^ix^—O15—B9—O14	171.4 (3)
B8^ii^—K2—O3—B6	−113.1 (2)	B10—O15—B9—O17	−177.1 (3)
K1^vii^—K2—O3—B6	89.4 (2)	K1^ix^—O15—B9—O17	−4.9 (3)
K1—K2—O3—B6	−160.0 (2)	B10—O15—B9—K1^ix^	−172.2 (3)
O9^v^—K2—O3—B2	130.2 (2)	B10—O15—B9—K1^ii^	−68.3 (3)
O14^ii^—K2—O3—B2	60.3 (2)	K1^ix^—O15—B9—K1^ii^	103.98 (19)
O4^iii^—K2—O3—B2	−39.5 (2)	B8—O16—B10—O18	175.3 (3)
O8^iii^—K2—O3—B2	0.9 (2)	B8—O16—B10—O15	−6.3 (5)
O12^ii^—K2—O3—B2	97.0 (2)	B9—O15—B10—O16	1.8 (5)
O7^v^—K2—O3—B2	−116.4 (3)	K1^ix^—O15—B10—O16	−165.4 (2)
O13^vi^—K2—O3—B2	−120.6 (2)	B9—O15—B10—O18	−179.8 (3)
B4^v^—K2—O3—B2	141.9 (3)	K1^ix^—O15—B10—O18	13.0 (5)

Symmetry codes: (i) *x*−1, *y*−1, *z*−1; (ii) −*x*+1, −*y*+1, −*z*+1; (iii) −*x*+1, −*y*, −*z*+1; (iv) −*x*, −*y*, −*z*+1; (v) *x*, *y*, *z*−1; (vi) −*x*+2, −*y*+1, −*z*+1; (vii) *x*+1, *y*, *z*; (viii) *x*, *y*, *z*+1; (ix) *x*+1, *y*+1, *z*+1; (x) *x*, *y*+1, *z*; (xi) *x*, *y*−1, *z*.

### Hydrogen-bond geometry (Å, °)

**Table:**

*D*—H···*A*	*D*—H	H···*A*	*D*···*A*	*D*—H···*A*
O1—H1···O11^xii^	0.88 (2)	1.76 (2)	2.599 (3)	159 (3)
O9—H9···O18^xiii^	0.87 (2)	1.98 (2)	2.797 (3)	157 (3)
O11—H11···O19^xiv^	0.92 (2)	1.65 (2)	2.553 (3)	167 (3)
O18—H18···O5^xv^	0.89 (2)	2.10 (2)	2.940 (3)	157 (3)
O18—H18···O12^xv^	0.89 (2)	2.66 (3)	3.193 (3)	119 (3)
O19—H19A···O6^iv^	0.90 (2)	1.79 (3)	2.678 (3)	169 (3)
O19—H19B···O16^iii^	0.91 (2)	1.79 (3)	2.696 (3)	173 (3)

Symmetry codes: (xii) *x*−1, *y*−1, *z*; (xiii) *x*−1, *y*, *z*; (xiv) *x*+1, *y*+1, *z*; (xv) −*x*+2, −*y*+1, −*z*+2; (iv) −*x*, −*y*, −*z*+1; (iii) −*x*+1, −*y*, −*z*+1.
